# L’évaluation du syndrome du Burnout chez les médecins en formation au CHU Ibn Rochd de Casablanca

**DOI:** 10.11604/pamj.2017.27.243.6257

**Published:** 2017-08-02

**Authors:** Assiya El Kettani, Zineb Serhier, Mohammed Bennani Othmani, Mohamed Agoub, Omar Battas

**Affiliations:** 1 Laboratoire d’Informatique Médicale, Faculté de Médecine et de Pharmacie de Casablanca, Maroc; 2 Laboratoire de Neurosciences Cliniques et Santé Mentale, Faculté de Médecine et de Pharmacie de Casablanca, Maroc

**Keywords:** Burnout, médecins en formation, CHU Ibn Rochd, Burnout, doctors in training, Ibn Rochd University Hospital

## Abstract

**Introduction:**

Le Burnout est très répandu en milieu hospitalier et sa gravité est encore plus accrue chez les jeunes médecins. L'objectif est de déterminer la prévalence du burnout parmi les médecins en formation (internes et résidents) au CHU Ibn Rochd de Casablanca et chercher les facteurs associés.

**Méthodes:**

Étude transversale descriptive menée en 2013- 2014 auprès d'un échantillon de 300 médecins à l'aide d'un questionnaire auto-administré; le burnout était évalué par la version française du Maslash Burnout Inventory (MBI).

**Résultats:**

Un total de191 médecins a participé à l'étude (taux de réponse à 63,7%) avec une prédominance féminine à 79,1% et une moyenne d'âge de 26,7ans (ET = 3). Les scores moyens des sous-dimensions du MBI: l'épuisement émotionnel, la dépersonnalisation et l'accomplissement personnel étaient respectivement de (33,7 ± 10,7), (12,2 ± 6,5) et (30,6 ± 8,3). Le burnout sévère touchait 31,8% des participants. Il était associé aux problèmes de communication au sein de l'équipe soignante (p < 0,01), l'accompagnement insuffisant (p < 0,05), l'insatisfaction des séniors (p = 0,01), la crainte de faire des erreurs médicales (p < 0,05), le recours à un psychothérapeute (p < 0,001), la consommation de psychotropes (p = 0,001), les troubles anxieux (p < 0,01), la dépression (p < 0,01) et les idées suicidaires (p < 0,05). Les facteurs protecteurs étaient représentés par: le sentiment d'équité au sein de l'équipe soignante (p < 0,01) et la pratique de loisirs (p < 0,05). Le changement d'orientation de carrière était associé au burnout sévère (p < 0,05).

**Conclusion:**

Ces résultats rejoignent ceux des études précédentes et justifient l'intérêt d'un programme de prévention à différents niveaux.

**Introduction:**

Burnout is very widespread in the hospital setting and it becomes much more severe in young physicians. The objective of this study aims to evaluate the prevalence of burnout among doctors in training (internal and residents) at the Ibn Rochd University Hospital, Casablanca and to identify factors associated with it.

## Abstract (en)

**Methods:**

We conducted a descriptive cross-sectional study of a sample of 300 physicians using self-administered questionnaire over the period 2013- 2014. Burnout has been operationally defined through a French translation of the Maslach Burnout Inventory (MBI).

**Results:**

A total of 191 physicians participated in the study (response rate 63.7%) with a female predominance at 79.1% and an average age of 26.7 years (N = 3). The average scores of sub-dimensions of the MBI: emotional exhaustion, depersonalization and personal fulfilment were (33.7±10.7), (12.2±6.5) and (30.6±8.3) respectively. 31.8% of participants suffered from severe burnout. It was associated with communication problems within the healthcare team (p < 0.01), insufficient accompaniment (p < 0.05), dissatisfaction among seniors (p = 0.01), fear of making medical errors (p<0.05), use of a psychotherapist (p<0.001), use of psychotropic drugs (p = 0.001), anxiety disorders (p < 0.01), depression (p < 0.01) and suicidal ideation (p < 0.05). Protective factors were: sense of equity within the healthcare team (p < 0.01) and practice of leisure (p<0.05). Changing career direction was associated with severe burnout (p<0.05).

**Conclusion:**

These results are consistent with those of previous studies and justify the importance of a prevention program at different levels.

## Introduction

Le burnout (BO) est un syndrome psychologique susceptible d'apparaître chez des individus qui travaillent avec autrui, en réponse à l'accumulation de stresseurs émotionnels et interpersonnels au travail [1]. Il résulte d'une inadéquation entre les attentes et la réalité professionnelle et il est auto-entretenu par des stratégies de coping inadéquates [2]. Selon Maslach et Jackson, il comprend 3 dimensions: **l'épuisement émotionnel (EE):** se réfère à la sensation d'être vidé de ses ressources émotionnelles et physiques et conduit à des difficultés à être en relation avec les émotions de l'autre. Il représente la réponse basique au stress; **la dépersonnalisation (DP):** représente une réponse négative ou excessivement détachée envers d'autres personnes qui sont considérées comme des objets. Elle est utilisée comme une stratégie de coping pour gérer l'épuisement; **la perte de l'accomplissement personnel (AP):** fait référence aux sentiments d'incompétence et d'inefficacité, de manque de réussite et de productivité au travail. Elle représente la dimension d'auto-évaluation du burnout.

Le burnout est très répandu parmi les médecins [1] et sa gravité est encore plus accrue chez les jeunes médecins. Environ le quart des jeunes médecins sont atteints de dépression et plus de 10% ont des idées suicidaires [3]. Outre son retentissement sur la qualité de vie de ceux qui en souffrent et la perception de leur profession, le burnout a un impact négatif sur la qualité des soins délivrés (erreurs médicales, prises de décisions inappropriées) [4]. Dans notre contexte seules quelques études ont été réalisées; d'où les objectifs de ce travail étaient de déterminer la prévalence du burnout parmi les médecins en formation au CHU Ibn Rochd de Casablanca et de chercher les facteurs associés.

## Méthodes

Il s'agit d'une étude transversale descriptive menée en 2013/2014. La population source était constituée des médecins en formation (internes et résidents) du CHU Ibn Rochd de Casablanca. L'étude avait porté sur un échantillon obtenu par un sondage stratifié en grappe. Nous avons exclu les médecins n'exerçant aux services n'offrant pas de prestations de soins et d'hospitalisation : la radiologie, la médecine nucléaire, la biologie, l'anatomo-pathologie, la génétique, la médecine de travail, la médecine légale, la médecine communautaire et l'informatique médicale. Le critère de stratification était la nature et le pronostic des maladies traitées ; ainsi les strates étaient : Strate 1: les services traitant des pathologies lourdes ou à pronostic vital élevé: les services d'anesthésie-réanimation, le service des brulés, l'onco-hématologie, la cardiologie, les maladies infectieuses [5]; Strate 2: les services de chirurgie; Strate 3: les services de médecine; Strate 4: les services de pédiatrie; Strate 5: les services de gynécologie; Strate 6: les services de psychiatrie et de pédopsychiatrie; Strate 7: les services de médecine dentaire. Les grappes au sein des strates étaient: strate 1: l'anesthésie-réanimation, maladies infectieuses; strate 2: l'ophtalmologie, la chirurgie pédiatrique; strate 3: la gastrologie, la médecine interne, la néphrologie, la dermatologie; strate 4: le service d'accueil des urgences pédiatriques, le service de néonatologie ; strate 5: le plateau technique de gynécologie ; strate 6: le service de psychiatrie; strate 7: le centre de soins dentaires. La collecte des données était réalisée à l'aide d'un questionnaire auto-administré comportant cinq sections: données sociodémographiques et professionnelles; la version française du Maslach Burnout Inventory (MBI-HSS); les facteurs de stress hospitaliers perçus et des données sur l'état de santé des médecins et le questionnaire de Cherniss des orientations de carrière ou identité professionnelle.

Le MBI comporte 22 items dont les réponses sont quottées par une échelle Likert de 0 (jamais) à 7 (chaque jour). Il mesure les 3 dimensions du burnout: l'épuisement émotionnel, la dépersonnalisation et l'accomplissement personnel. Le calcul des scores est obtenu par la somme des réponses aux items. Les niveaux du burnout (élevé, moyen, bas) sont définis par des seuils spécifiques: EE (BO bas: score < 18, BO modéré: 18 à 29, BO élevé: > 29); DP (BO bas: < 6, BO modéré: 6 à 11, BO élevé: > 11); AP (BO élevé: < 34, BO modéré: 34 à 39, BO bas: > 39). Dans cette étude, nous avons considéré que le burnout est sévère quand les deux premiers scores sont élevés et le troisième est bas. Les associations avec le burnout sévère ont été établies avec le test de khi-deux (variables qualitatives) et par l'analyse de variance (variables quantitatives) nous avons également réalisé une analyse multivariée par une régression logistique (méthode pas à pas descendante). L'analyse a été réalisée par le logiciel SPSS 16.

## Résultats

**Figure 1:**
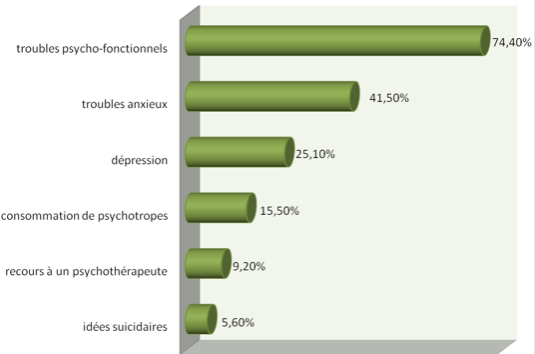
Les problèmes de santé rapportés par les médecins

**Figure 2:**
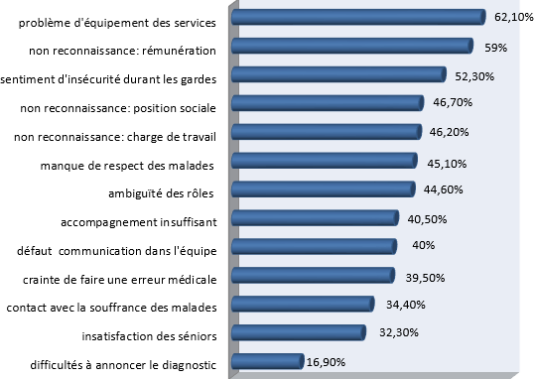
Les facteurs de stress hospitaliers perçus par les médecins

**Figure 3:**
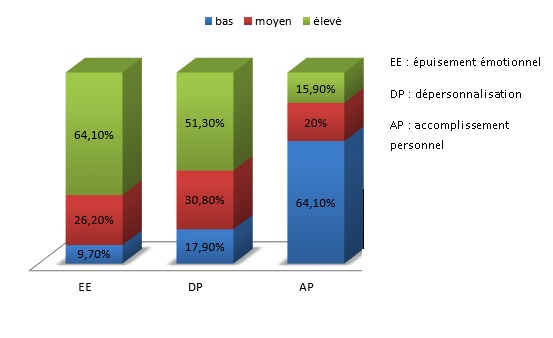
La répartition des niveaux des sous-dimensions du burnout chez les participants

Sur 300 questionnaires distribués, 191 ont été retenus soit un taux de réponses de 63,7 %. Le questionnaire de Cherniss était, par contre, complété par 151 personnes (soit un taux de réponses de 50,3%). Il y avait une prédominance féminine de 79,1%, l?âge moyen était de 26,7 ans (ET= 3), 34,9% des participants étaient mariés et 19,20% avaient des enfants à charge. En ce qui concerne la charge de travail, la moyenne des nombres d'heures travaillées par semaine était de 43,14 h (ET = 15,84) les services d'anesthésie-réanimation, hémato-oncologie, cardiologie et maladies infectieuses avaient le plus grand nombre d'heures travaillées par semaine (53,82h (ET=17,99)), la moitié des médecins passaient plus de 3 gardes par mois et 80 % ont eu moins de 2 semaines de vacances au cours du semestre où s'est déroulée l'étude. Environ 27,0% ont déclaré consacrer suffisamment de temps à l'entourage, 23,6% pratiquaient régulièrement un loisir et 53,8% trouvaient que l'internat/résidanat est un obstacle à la maternité/paternité. Par ailleurs, 2,1% étaient tabagiques et 2,6% consommaient de l'alcool. Les problèmes de santé les plus rapportés par les médecins (depuis le début de leur internat/résidanat) étaient les troubles psycho-fonctionnels (ex : lombalgies, épigastralgies) avec une fréquence de 74,4%, les troubles anxieux avec une fréquence de 41,5% et la dépression avec une fréquence de 25,1% (Figure 1). Les causes du stress hospitalier perçues par les participants étaient essentiellement : le problème d'équipement des services (62,1%), la non reconnaissance de l'interne/résident à sa juste valeur quant à la rémunération (59,0%), le sentiment d'insécurité durant les gardes (52,3%) (Figure 2). Les scores moyens de l'épuisement émotionnel, de la dépersonnalisation et de l'accomplissement personnel étaient respectivement : 33,7(ET : 10,7), 12,2 (ET : 6,5), 30,6 (ET : 8,3). A niveau élevé, l'(EE) touchait 63,5% des participants, la (DP) 55,6% et la diminution de l'(AP) 73,0% (Figure 3). Le burnout sévère était noté chez 31,8% des participants.

En ce qui concerne les orientations de carrière des médecins, à l'entrée de la profession les artisans prédominaient (43%) et étaient suivis des activistes (32%). Par la suite les activistes ont diminué (17% vs 32%) et les égoïstes sont devenus plus nombreux (13% vs 2%). Environ 60,0% ont changé d'orientation. Le Tableau 1 résume les détails du passage de l'orientation de carrière initiale à l'orientation au moment de l'étude. Les facteurs associés significativement à l'épuisement émotionnel, à la dépersonnalisation ou à la diminution de l'accomplissement personnel étaient: les problèmes d'équipement des services (p < 0,001); le sentiment d'insécurité durant les gardes (p < 0,01); l'accompagnement insuffisant (p < 0,05); l'ambiguïté des rôles (p < 0,05); la non reconnaissance de l'interne/résident à sa juste valeur quant à la charge de travail (p < 0,05); l'insatisfaction des séniors (p = 0,01); le défaut de communication entre les membres de l'équipe soignante (p < 0,01); les difficultés de la relation médecin-malade : manque de respect des patients vis-vis des médecins (p < 0,05), difficultés à communiquer les informations et à annoncer le diagnostic au patient et à sa famille (p < 0,01); le conflit entre la vie professionnelle et la vie privée : penser que l'internat/résidanat est un obstacle à la maternité/paternité (p < 0,05), les problèmes de somatisations (p < 0,01); le recours à un psychothérapeute (p < 0,05); les troubles anxieux et dépressifs (p<0,05).

**Tableau 1:** Les détails du passage de l’orientation de carrière initiale à l’orientation de carrière au moment de l’étude

Orientation initiale	Orientation au moment de l’étude			
	Activiste	Artisan	Carriériste	Égoïste
Activiste				
N:	12	19	9	7
%	25,6	40,4	19,1	14,9
Artisan				
N:	5	37	19	5
%	7,6	56,0	28,8	7,6
Carriériste				
N:	7	11	10	7
%	20,0	31,4	28,6	20,0
Égoïste				
N:	1	1	0	1
%	33,3	33,3	0,0	33,3

Les facteurs protecteurs étaient: le sentiment d'équité au sein de l'équipe soignante (p = 0,01); le temps consacré à l'entourage (p < 0,01); et la pratique de loisirs (p = 0,001) (Tableau 2, Tableau 3, Tableau 4).

**Tableau 2:** Les facteurs associés à l’épuisement émotionnel (EE)

Facteurs	EE bas	EE modéré	EE élevé	*p*-value
**Problèmes d’équipement**				
Non	15,6	45,3	39,1	<0,001
Oui	4,0	75,2	20,8	
**Ambiguïté des rôles**				
Non	12,2	58,2	29,6	0,042
Oui	3,4	72,4	24,2	
**Problèmes de communication**				
Non	12,4	56,2	31,4	0,009
Oui	2,5	75,6	21,9	
**Absence d’accompagnement**				
Non	1,3	82,3	16,4	0,018
Oui	9,5	62,0	28,5	
**Insatisfaction des séniors**				
Non	10,8	58,3	30,9	0.024
Oui	3,2	77,8	19,0	
**Temps consacré à l’entourage**				
Non	3,2	81,7	15,1	0,007
Oui	7,4	51,8	40,8	
**Pratique de loisirs**				
Non	6,2	71,5	22,3	0,001
Oui	17,4	41,3	41,3	
**Facteurs**	**EE bas**	**EE modéré**	**EE sévère**	**p-value**
**Problèmes de somatisations**				
Non	14,3	45,2	40,5	0,007
Oui	6,2	71,0	22,8	
**Troubles anxieux**				
Non	10,4	56,6	33,0	0,017
Oui	4,9	76,5	18,6	
**Dépression**				
Non	9,4	59,4	31,2	0,020
Oui	4,0	81,6	14,4	

**Tableau 3:** Les facteurs associés à la dépersonnalisation (DP)

Facteurs	DP basse	DP modérée	DP élevée	p-value
**problèmes de communication**				
Non	2,0	42,8	55,2	**0.026**
Oui	11,5	62,8	25,7	
**Absence d’accompagnement**				
Non	28,6	33,3	38,1	**0,003**
Oui	8,8	62,0	29,2	
**Manque de respect des patients vis-à-vis des médecins**				
Non	28,1	34,4	37,5	**0,025**
Oui	11,4	59,1	29,5	
**Penser que le travail est un Obstacle à la maternité/paternité**				
Non	15,2	80,9	3,9	**0,013**
Oui	18,8	41,2	40,0	
**Sentiment d’équité dans l’équipe**				
Non	11,1	58,7	30,2	**0.001**
Oui	27,7	31,5	40,8	
**Pratique de loisirs**				
Non	15,5	55,6	28,9	**0,001**
Oui	19,6	41,3	39,1	
**Facteurs**	**DP basse**	**DP modérée**	**DP élevée**	***p*-value**
**Troubles anxieux**				
Non	18,3	46,7	35,0	**0,024**
Oui	15,0	54,3	30,7	
**Dépression**				
Non	18,8	47,1	34,1	**0,009**
Oui	8,2	65,3	26,5	
**Recours à un psychothérapeute**				
Non	17,1	48,5	34,4	**0,019**
Oui	5,5	83,3	11,2	

**Tableau 4:** Les facteurs associés à l’accomplissement personnel (AP)

Facteurs	AP bas	AP modéré	AP élevé	p-value
**Sentiment d’insécurité durant les gardes**				
Non	62,7	19,3	18,1	0.004
Oui	62,7	14,7	22,5	
**Difficultés à communiquer les informations au patient et à sa**				
**Famille**				
Non	61,8	17,1	21,1	0,005
Oui	66,7	12,1	21,2	
**Non reconnaissance de la Charge de travail**				
Non	66,7	11,1	22,2	0,020
Oui	35,5	32,3	32,3	
**Penser que le travail est un Obstacle à la maternité/paternité**				
Non	52,4	25,0	22,6	0,009
Oui	71,4	9,5	19,0	
**Recours à un psychothérapeute**				
Non	59,8	17,1	23,1	0,035
Oui	88,9	11,1	0,0	

Les facteurs associés significativement au burnout sévère étaient: les problèmes de communication au sein de l'équipe soignante (p < 0,01); l'accompagnement insuffisant (p<0,05); l'insatisfaction des séniors (p=0,01); la crainte de faire des erreurs médicales (p<0,05); le recours à un psychothérapeute (p<0,001); les troubles anxieux (p = 0,01); la dépression (p < 0,01); les idées suicidaires (p < 0,05).

Les facteurs protecteurs étaient représentés par: le sentiment d'équité au sein de l'équipe soignante (p < 0,01); et la pratique de loisirs (p < 0,05) (Tableau 5).

**Tableau 5:** Les facteurs associés au burnout sévère

Facteurs	Burnout sévère (%)	p-value
**Défaut de communication au sein de l’équipe soignante**		
Non	22,9	0,008
Oui	41,0	
**Absence d’accompagnement**		
Non	21,4	0,025
Oui	41,8	
**Insatisfaction des séniors**		
Non	25,8	0,010
Oui	44,5	
**Crainte de faire des erreurs médicales**		
Non	30,0	0,018
Oui	57,2	
**Sentiment d’équité**		
Non	36,5	0.004
Oui	14,8	
**Facteurs**	**Burnout severe (%)**	**p-value**
**Consommation de psychotropes**		
Non	26,6	0,001
Oui	58,6	
**Recours à un psychothérapeute**		
Non	27,2	<0,001
Oui	72,2	
**Troubles anxieux**		
Non	22,7	0,003
Oui	59,3	
**Dépression**		
Non	26,0	0,007
Oui	47,0	
**Idées suicidaires**		
Non	29,6	0,024
Oui	63,6	

En analyse multivariée, les facteurs qui ont été retenus comme étant indépendamment associés au burnout sévère étaient: le défaut de communication au sein de l'équipe soignante: OR = 5,54 ; IC95% (1,78; 17,22); p = 0,003.

La crainte de faire des erreurs médicales: OR = 4,76; IC95% (1,13; 19,99); p = 0,033.

Le sentiment d'équité au sein de l'équipe soignante: OR = 0,20; IC95% (0,05; 0,78); p = 0,021.

Le recours à un psychothérapeute: OR= 7,75; IC95% (1,27; 47,22); p = 0,026.

Les autres facteurs qui n'étaient pas significativement associés au burnout (p>0,05) étaient: l'âge, le sexe, la situation familiale et le nombre d'enfants à charge, l'année d'exercice, le service d'exercice, la charge de travail, le contact avec la souffrance des malades, la non reconnaissance: position sociale, rémunération, et la consommation du tabac et de l'alcool.

En ce qui concerne les orientations de carrière au moment de l'étude et le burnout, les activistes avaient l'épuisement émotionnel le plus bas (m = 32,7) très proche de celui des artisans (m = 32,8), Ils étaient ceux qui dépersonnalisaient le moins leurs patients (m = 11,4) et étaient les plus accomplis (m = 32,7). Les carriéristes avaient l'épuisement émotionnel (m = 35,7) et la dépersonnalisation (m=14,0) les plus élevés. Leur accomplissement personnel (m = 31,3) faisait suite à celui des activistes. Les artisans avaient un EE (m = 32,8) et une DP (m = 13,8) plus bas que ceux des carriéristes mais un AP (m = 31,5) plus bas aussi. Les égoïstes avaient un EE (m=33,7) plus élevé que celui des artisans, une DP (m = 12,2) plus basse mais un AP (m = 30,6) plus bas que les activistes et les carriéristes; cependant ces associations n'étaient pas statistiquement significatifs (Tableau 6). Le changement d'orientation était par ailleurs lié au burnout sévère (p=0,014).

**Tableau 6:** Les orientations de carrière des médecins au moment de l’étude et le burnout

Scores moyens (ET)	Orientation de carrière au moment de l’étude				
	Activiste	Artisan	Carriériste	Égoïste	p-value
Epuisement émotionnel	32,7 (10,9)	32,8 (10,8)	35,7 (10,7)	33,7 (10,0)	0,68
Dépersonnalisation	11,4 (6,3)	13,8 (6,2)	14,0 (7,0)	12,2 (4,9)	0,06
Accomplissement personnel	32,3 (8,2)	29,4 (8,0)	31,3 (7,4)	30,6 (8,0)	0,17

## Discussion

Parmi les 300 personnes interrogées, 191 (taux de réponses de 63,7 %) ont rempli le questionnaire. Nous avons noté une prévalence élevée du burnout parmi les médecins en formation du CHU Ibn Rochd (burnout sévère: 31,8%). Plusieurs facteurs y étaient associés et étaient d'ordres: organisationnel: les problèmes d'équipement des services hospitaliers, l'ambiguïté des rôles, le sentiment d'insécurité durant les gardes, l'absence d'accompagnement, la crainte de faire des erreurs médicales, le conflit entre vie professionnelle et vie privée; interindividuel: l'insatisfaction des séniors, le défaut de communication entre les membres de l'équipe soignante, les difficultés de la relation médecin/malade: manque de respect, difficultés à annoncer le diagnostic et à communiquer les informations au patient et à sa famille.

Les facteurs protecteurs étaient: le sentiment d'équité au sein de l'équipe soignante, le temps consacré à l'entourage et la pratique de loisirs. Des conséquences ont été relevées avec une prévalence considérable des maladies psycho-fonctionnelles, des troubles anxieux et de la dépression. Le changement d'orientation de carrière était associé au burnout. Les propriétés psychométriques de la version française du MBI sont satisfaisantes; (α de Cronbach des sous dimensions: EE = 0,90, DP = 0,64, AP = 0,74) [6]. Le type d'échantillonnage probabiliste (stratifié en grappe) et le taux de réponse relativement moyen avaient permis de réduire l'existence d'un biais de sélection. Par contre, les données transversales de l'étude ne peuvent conclure à une relation de cause à effet entre les différents facteurs et le burnout. Les scores moyens des sous-dimensions du burnout de notre étude étaient globalement comparables aux scores d'une étude publiée en 2006 et réalisée auprès de médecins en formation à Minnesota (US), d'une étude publiée en 2008 et réalisée auprès de médecins généralistes dans 12 pays européens ainsi que d'étude publiée en 2000 et réalisée au CHU Ibn Rochd dans 5 unités prenant en charge des pathologies lourdes (Réanimation, Hématologie, Oncologie, maladies infectieuses, brûlés). Elles avaient également enregistré des niveaux modérés à élevés des trois sous-dimensions [7–9]. L'étude publiée en 2009 et réalisée auprès du personnel de la réanimation dans 4 CHU marocains avait également rapporté un score moyen élevé de l'épuisement émotionnel et de la diminution de l'accomplissement personnel et un score modéré de la dépersonnalisation [10]. Par contre, Ils discordent avec les scores moyens d'une étude publiée en 2013 et réalisée en France, auprès des internes en médecine générale, qui avait noté des scores modérés des 3 sous-dimensions (Tableau 7) avec cependant, une prévalence de burnout sévère légèrement plus faible que la nôtre (24,1%) [11] ; ceci pourrait être en rapport avec des mesures préventives qui ont été déjà instaurées. De même, nous n'avons pas noté d'association entre l'épuisement professionnel et les variables sociodémographiques comme la situation familiale et le nombre d'enfants à charge. Le rôle du sexe est controversé, probablement en rapport avec le fait que les femmes, dans certains cas, cumulent d'autres facteurs de stress extraprofessionnels et la difficulté à concilier les contraintes de travail et les charges familiales. L'âge nécessite une discussion particulière. Il semble qu'il existe une relation entre l'âge et les deux dimensions de l'épuisement professionnel: l'épuisement émotionnel et la déshumanisation de la relation. Les personnes les plus jeunes en sont plus vulnérables.

**Tableau 7:** Comparaison de nos résultats avec ceux de la littérature

Etude	Lieu	Population de l’étude	Nombre de participants	Taux de réponses (%)	EE DP AP modérés à élevés (%)			EE DP AP Scores moyens		
Dyrbye, 2006 (7)	Minnesota (US)	Médecins en formation	545	50,0	62,5	48,2	69,2	34,4	26,9	28,2
EGPRN, 2008 (8)	12 pays Européens	Médecins généralistes	1393	41,0	83,0	62,5	60,0	43,0	35,0	32,0
Galam, 2013 (11)	France Métropolitaine	Médecins en formation	4050	71,0	56,9	77,3	72,5	20,0	9,7	34,8
Massou, 2013 (10)	4 CHU Marocains	Personnel de réanimation	290	94,1	76,5	47,6	73,8	28,6	6,9	33,6
Agoub, 2000 (9)	Casablanca (Maroc)	Personnel de 5 unités traitant des pathologies lourdes	68	75,5	80,1	73,5	42,6	28,0	10,9	30,3
Notre étude	Casablanca (Maroc)	Médecins en formation	191	63,7	90,4	82,1	64,1	33,7	12,2	30,6

EGPRN: European General Practice Research Network.

EE: Epuisement émotionnel, DP: Dépersonnalisation, AP: Accomplissement personnel

Le facteur charge de travail est également sujet à des controverses. A charge égale, un médecin reconnu et valorisé serait moins épuisé. En plus, l'impact des conditions de travail serait plus important que la charge de travail à elle seule. D'autre part, si une surcharge de travail peut être nocive pour la santé mentale des travailleurs, il en est de même pour une sous-charge suscitant de l'ennui et une baisse de la motivation et de la satisfaction au travail. Par ailleurs, des enquêtes ont démontré que les heures excessives de travail n'engendraient pas nécessairement une augmentation de la productivité. Elles auraient plutôt tendance à diminuer l'efficacité et l'efficience des individus ; et il se pourrait que les soignants souffrant de burnout augmentent leur charge de travail pour palier au sentiment d'échec [1, 7, 12, 13]. Une partie de la souffrance des soignants provient de la discordance entre l'utopie de l'état de santé parfait et la réalité de l'homme souffrant , de la confrontation quotidienne avec les limites de l'efficacité médicale et du malaise des médecins face aux attentes irréalistes de la société vis-à-vis d'un établissement de soins de 3ème ligne qu'est le centre hospitalier universitaire (dans notre contexte) [14].

Par contre, la détresse que génère la confrontation avec la souffrance des malades, à elle seule, n'était que marginalement citée dans les causes de stress indiquées (34,4%). Elle n'était pas significativement associée au burnout et il n'y avait pas, non plus, de différence significative entre les scores obtenus selon les différents services concernés par l'étude. Les médecins ont insisté plutôt sur les sources organisationnelles et interindividuelles. Les problèmes de santé relevés ont été rapportés et peuvent faire l'objet d'une sous-estimation ou encore d'une surestimation. D'autant plus que les médecins ont un comportement particulier envers leur propre santé : difficulté à demander de l'aide, l'autodiagnostic et l'automédication [15]. Cependant, se basant sur les symptômes dépressifs dans la diminution de l'accomplissement personnel, des études ont rapportées que le syndrome d'épuisement professionnel peut faire le lit d'une dépression majeure, et conduire même au suicide [16]. Les troubles psycho-fonctionnels étaient les problèmes les plus évoqués par les médecins. Des auteurs ont conclu à une relation significative entre les signes physiques et le burnout. Ils ont noté une sensibilité accrue des victimes de l'épuisement professionnel aux troubles psychosomatiques et psycho-fonctionnels [17]. En outre, le brun out s'associe de manière significative à des conduites à risque pour les médecins et leurs malades (consommation excessive d'alcool, prise de psychotropes) la discordance relevée dans notre étude pourrait être due à un manque de puissance statistique [11].

Pour ce qui est des orientations de carrière, chaque individu entretient des attentes particulières vis-à-vis de son travail : faire vivre certaines valeurs auxquelles il est attaché, renforcer son sentiment d'auto-efficacité, son estime personnelle ou simplement son confort matériel. Cependant on peut observer un réajustement des aspirations initiales, passant d'une orientation tournée vers le social et le plaisir à exercer son activité à un repli sur la sphère privée et ceci peut traduire des problématiques individuelles [18]. Selon l'étude de Truchot, les artisans avaient l'épuisement professionnel le plus bas et constituaient un groupe protégé du burnout, les activistes avaient un burnout faible avec un accomplissement personnel élevé, les carriéristes étaient les plus stressés et les égoïstes les moins accomplis. Nous avons observé une large tendance à se replier sur l'orientation égoïste. Mais plutôt qu'un choix motivé par la recherche d'un mieux-être, il semble qu'il s'agit d'une sorte d'option par défaut et qui n'est pas la meilleure stratégie pour se prémunir du burnout (EE = 33,7 (élevé)), (DP = 12,2 (élevée)), (AP = 30,6 (bas)). Elle guette, selon Truchot les jeunes médecins [19].

Nous n'avons pas évalué l'impact négatif du burnout sur les performances médicales, mais cet impact est vraisemblablement significatif; la dépersonnalisation est souvent ressentie comme une forme d'échec personnel qui va être exprimé par la diminution de l'accomplissement professionnel [1]. En plus, Si le médecin en début de carrière n'éprouve pas de satisfaction quant à son accomplissement professionnel, il serait difficile de penser qu'il s'investira par la suite dans le bon fonctionnement du système de soins.

Dans notre contexte, tout programme de prévention s'articulerait autour de 3 axes : Prévention primaire: définir la part institutionnelle, administrative et juridique dans l'approche de l'ergonomie du travail à l'hôpital, passer de l'enseignement classique à une formation individualisée permettant un accompagnement personnalisé et un tutorat. Prévention secondaire: améliorer de la communication, apprendre à travailler en équipe, adopter d'une une meilleure hygiène de vie. Prévention tertiaire : reconnaître le burnout comme maladie professionnelle, l'inclure dans les classifications nosographiques et en proposer une prise en charge codifiée.

Dans le cadre de la poursuite de notre travail, il serait pertinent d'étendre l'étude aux autres médecins en formation des autres CHU du pays, de le compléter par des études à visée analytique; ensuite refaire l'état des lieux dans quelques années afin de juger de l'évolution du phénomène en fonction des changements effectués.

## Conclusion

Le burnout des médecins en formation au CHU Ibn Rochd est une réalité, d'autant plus que les résultats obtenus font partie des plus hauts niveaux de burnout décrits dans la littérature. Ceci retentit non seulement sur leur qualité de vie et la perception de leur profession mais également sur la qualité des soins délivrés. Les facteurs associés étaient d'ordres organisationnels et interindividuels. D'où l'intérêt de mettre en œuvre un programme rationnel de prévention selon différents niveaux, afin de permettre une meilleure efficacité-qualité du travail à l'hôpital. Il serait également pertinent de discuter l'apport de la technologie de l'information dans la résolution des problèmes organisationnels de la pratique clinique (le système d'information hospitalier et la télémédecine à titre d'exemple).

### Etat des connaissances actuelle sur le sujet

- Le burnout est très répandu parmi les jeunes médecins dans le monde;
- Il résulte de l'accumulation de stresseurs professionnels et interpersonnels présents depuis un certain temps dans le milieu de travail;
- Il peut avoir des conséquences graves sur la qualité de vie de ceux qui en souffrent, sur la perception de leur profession mais aussi sur la qualité de soins délivrés.

### Contribution de notre étude à la connaissance

- Cette étude avait révélé que le niveau du burnout parmi les médecins en formation au CHU Ibn Rochd de Casablanca fait partie des plus hauts niveaux décrits dans la littérature;
- Les facteurs associés étaient d'ordres organisationnels et interindividuels;
- Cette étude pourrait offrir des pistes de travail pour un programme de prise en charge et de prévention mais aussi pour une comparaison avec d'autres CHU du pays et dans le monde.

## Conflits d’intérêts

Les auteurs ne déclarent aucun conflit d'intérêts.
